# Thermal equation of state of ruthenium characterized by resistively heated diamond anvil cell

**DOI:** 10.1038/s41598-019-51037-8

**Published:** 2019-10-08

**Authors:** Simone Anzellini, Daniel Errandonea, Claudio Cazorla, Simon MacLeod, Virginia Monteseguro, Silvia Boccato, Enrico Bandiello, Daniel Diaz Anichtchenko, Catalin Popescu, Christine M. Beavers

**Affiliations:** 1 Diamond Light Source Ltd., Harwell Science & Innovation Campus, Diamond House, Didcot, OX11 0DE UK; 2 Departamento de Física Aplicada - Instituto de Ciencia de Materiales, Matter at High Pressure (MALTA) Consolider Team, Universidad de Valencia, Edificio de Investigación, C/Dr. Moliner 50, Burjassot, 46100 Valencia, Spain; 3 School of Materials Science and Engineering, University of New South Wales Sydney, Sydney, New South Wales 2052 Australia; 4 AWE, Aldermaston, Reading, RG7 4PR United Kingdom; 5 SUPA, School of Physics and Astronomy, and Centre for Science at Extreme Conditions, The University of Edinburgh, Edinburgh, EH9 3FD United Kingdom; 6 Institut de Minéralogie, de Physique des Matériaux, et de Cosmochimie (IMPMC), Sorbonne Université - UPMC, UMR CNRS 7590, Muséum National d’Histoire Naturelle, IRD UMR 206, F-75005 Paris, France; 7 CELLS-ALBA Synchrotron Light Facility, 08290 Cerdanyola, Barcelona, Spain

**Keywords:** Physics, Condensed-matter physics

## Abstract

The high-pressure and high-temperature structural and chemical stability of ruthenium has been investigated via synchrotron X-ray diffraction using a resistively heated diamond anvil cell. In the present experiment, ruthenium remains stable in the hcp phase up to 150 GPa and 960 K. The thermal equation of state has been determined based upon the data collected following four different isotherms. A quasi-hydrostatic equation of state at ambient temperature has also been characterized up to 150 GPa. The measured equation of state and structural parameters have been compared to the results of *ab initio* simulations performed with several exchange-correlation functionals. The agreement between theory and experiments is generally quite good. Phonon calculations were also carried out to show that hcp ruthenium is not only structurally but also dynamically stable up to extreme pressures. These calculations also allow the pressure dependence of the Raman-active *E*_2*g*_ mode and the silent *B*_1*g*_ mode of Ru to be determined.

## Introduction

Transition metals have always attracted the interest of the scientific community due to their unusual electronic and structural properties, originating from the dominant influence of their *d* electrons. Often, they exhibit pronounced phonon anomalies as a result of complex Fermi-surface geometries coupled with strong electron-phonon interactions^1,2^. For these reasons, in the past decades big effort has been devoted to map and interpret the systematic properties of this metals at both ambient and extreme conditions of pressure and temperature^3^. Ruthenium (Ru) is a 4d transition metal that belongs to the platinum (Pt) group of the periodic table, along with rhodium (Rh), palladium (Pd), osmium (Os), iridium (Ir) and Pt^4^. Unlike the other elements of the group, Ru presents only one electron in the outermost shell.

At ambient pressure, Ru has a hexagonal close packed structure (*hcp*), melts at 2523 K, and becomes superconductor at temperatures below *T*_*c*_ = 0.47 K. In nature, it is generally found as a minor component in Pt ores. Ru is inert to most chemicals and it is generally used in wear-resistant electrical contacts and thick-film resistors^4–6^. In particular, it is often used alloyed with Pt and Pd as it increases their hardness. When alloyed with titanium (Ti), Ru improves its corrosion resistance, whereas, if alloyed with molibdenum (Mo) its *T*_*c*_ changes to 10.6 K^7^. Finally, Ru is also used as a chemical catalyst^8–10^.

Ru alloys have been extensively studied at extreme conditions (experimentally and theoretically) to investigate possible ultra-hard materials^11–13^.

In contrast with other 4d transition metals, like Mo and rhenium (Re)^14–18^, to the best of our knowledge, only few studies have been focused their attention on the characterization of pure Ru under extreme conditions. In particular, a study was performed in 1965 by Bucher *et al*.^19^ to characterize the effect of pressure on the superconductivity of Ru. The compression curve of Ru was experimentally measured up to 25 GPa by Clendenen *et al*.^20^ in a pressure-cylinder apparatus^21^. More recently, Cynn *et al*.^22^ studied Ru up to 56 GPa in diamond anvil cell (DAC), using argon (Ar) as pressure transmitting medium and energy-dispersive X-ray diffraction (ED-XRD) technique. In contrast with the results reported by Cynn *et al*., conclusions extracted from studies on iron-ruthenium alloys under high pressure suggest that a *hcp-fcc* transition should be expected in the range of the tenths of GPa^23^. On the other hand, a few theoretical works have been performed on *hcp* Ru. In one study, the stability field has been explored by means of elastic constant calculations^24^ and in a second study by first-principles calculations^25^. Information on the high-pressure and high-temperature behaviour of Ru is still lacking^5^; in particular, regarding its melting curve and thermal equation of state.

In this work, the structural stability of Ru has been investigated at room-temperature, up to 150 GPa by angular dispersive (AD) XRD, using helium as pressure medium. High-pressure (*HP*) high-temperature (*HT*) studies have also been performed to extend its structural and chemical characterization up to 975 K. The experiments have been combined with first-principle calculations of the structural and lattice dynamic properties.

## Results

### Evolution at ambient temperature

Two different experimental runs were carried out at ambient temperature at the extreme conditions beamline (I15) of Diamond Light Source under quasi-hydrostatic conditions. Table 1 reports the obtained results. During Run1 the pressure was increased from ambient up to 97.5 GPa with a maximum step of 2 GPa between consecutive pressures.

**Table 1:** The unit-cell parameters of Ru at ambient temperature as a function of pressure.

	a(Å)	c(Å)	c/a	V (Å^3^)	P (GPa)		a(Å)	c(Å)	c/a	V (Å^3^)	P (GPa)
**Run1**	2.705	4.281	1.583	27.141	0.00	**Run1**	2.552	4.052	1.588	22.856	78.4
	2.705	4.280	1.582	27.111	0.14		2.550	4.049	1.588	22.810	79.7
	2.704	4.280	1.583	27.107	0.33		2.549	4.047	1.588	22.768	81.2
	2.703	4.279	1.583	27.082	0.63		2.547	4.043	1.588	22.709	82.6
	2.701	4.276	1.583	27.022	1.36		2.545	4.040	1.588	22.600	83.9
	2.698	4.269	1.583	27.022	2.71		2.543	4.038	1.588	22.619	85.7
	2.696	4.266	1.583	26.903	3.40		2.541	4.035	1.588	22.572	87.6
	2.693	4.263	1.583	26.853	4.29		2.540	4.032	1.588	22.518	88.0
	2.691	4.259	1.583	26.779	5.29		2.537	4.029	1.588	22.463	89.9
	2.688	4.255	1.583	26.699	6.27		2.536	4.027	1.588	22.426	91.5
	2.685	4.251	1.583	26.626	7.48		2.534	4.024	1.588	22.383	92.3
	2.681	4.246	1.583	26.434	8.82		2.533	4.021	1.588	22.336	93.7
	2.677	4.239	1.584	26.303	10.4		2.531	4.019	1.588	22.297	95.0
	2.674	4.236	1.584	26.225	11.6		2.530	4.017	1.588	22.259	96.1
	2.671	4.232	1.584	26.154	12.6		2.528	4.015	1.588	22.223	97.5
	2.668	4.226	1.584	26.050	14.1	**Run2**	2.705	4.283	1.583	27.136	0.00
	2.664	4.220	1.584	25.937	15.6		2.705	4.280	1.582	27.112	0.20
	2.661	4.216	1.584	25.844	17.1		2.657	4.211	1.585	25.756	18.6
	2.656	4.209	1.585	25.710	18.9		2.612	4.145	1.587	24.495	40.7
	2.654	4.204	1.584	25.639	20.1		2.574	4.087	1.588	23.449	63.8
	2.650	4.200	1.585	25.550	21.6		2.557	4.063	1.589	23.005	75.1
	2.647	4.195	1.585	25.456	23.3		2.534	4.029	1.590	22.400	92.2
	2.643	4.190	1.585	25.351	24.8		2.530	4.024	1.590	22.308	95.1
	2.640	4.185	1.585	25.265	26.2		2.528	4.020	1.590	22.251	96.8
	2.637	4.181	1.585	25.183	27.6		2.525	4.016	1.590	22.177	99.1
	2.634	4.176	1.585	25.099	29.1		2.523	4.012	1.591	22.114	101
	2.632	4.172	1.585	25.026	30.5		2.520	4.008	1.591	22.036	104
	2.629	4.168	1.585	24.941	32.2		2.517	4.005	1.591	21.979	106
	2.626	4.164	1.586	24.866	33.4		2.515	4.001	1.591	21.919	107
	2.623	4.160	1.586	24.784	34.9		2.513	3.997	1.591	21.855	110
	2.621	4.156	1.586	24.719	36.2		2.510	3.994	1.591	21.800	111
	2.618	4.151	1.586	24.642	37.7		2.508	3.991	1.591	21.744	113
	2.616	4.148	1.586	24.578	39.0		2.506	3.988	1.591	21.691	115
	2.613	4.144	1.586	24.513	40.4		2.504	3.984	1.591	21.631	117
	2.611	4.140	1.585	24.448	41.7		2.502	3.982	1.591	21.585	119
	2.609	4.137	1.586	24.380	43.0		2.500	3.979	1.591	21.538	121
	2.606	4.133	1.586	24.305	44.6		2.498	3.976	1.592	21.492	122
	2.604	4.130	1.586	24.253	45.7		2.497	3.973	1.591	21.446	124
	2.600	4.124	1.586	24.149	47.4		2.494	3.970	1.592	21.393	126
	2.600	4.123	1.586	24.132	47.9		2.492	3.967	1.592	21.338	128
	2.599	4.124	1.587	24.115	48.6		2.490	3.964	1.592	21.288	130
	2.597	4.121	1.587	24.070	49.8		2.488	3.960	1.592	21.226	132
	2.595	4.119	1.587	24.023	50.7		2.485	3.956	1.592	21.164	135
	2.594	4.116	1.587	23.978	51.9		2.483	3.953	1.592	21.111	137
	2.591	4.113	1.587	23.922	53.2		2.481	3.950	1.592	21.058	139
	2.590	4.111	1.588	23.875	54.2		2.479	3.948	1.592	21.013	141
	2.588	4.108	1.587	23.827	55.2		2.477	3.944	1.592	20.957	143
	2.587	4.106	1.587	23.788	56.2		2.475	3.942	1.592	20.912	145
	2.585	4.104	1.588	23.745	57.1		2.473	3.939	1.593	20.861	147
	2.583	4.101	1.588	23.695	58.2		2.471	3.936	1.593	20.818	148
	2.582	4.098	1.588	23.653	59.1		2.469	3.933	1.593	20.769	151
**Run1**	2.580	4.096	1.588	23.613	60.2						
	2.578	4.094	1.588	23.565	60.9						
	2.577	4.091	1.588	23.527	61.9						
	2.576	4.089	1.588	23.490	62.1						
	2.574	4.088	1.588	23.459	63.8						
	2.573	4.085	1.588	23.416	65.0						
	2.570	4.080	1.588	23.333	66.6						
	2.568	4.076	1.587	23.270	68.4						
	2.565	4.074	1.588	23.216	70.0						
	2.562	4.070	1.588	23.151	71.5						
	2.560	4.066	1.588	23.083	73.2						
	2.558	4.063	1.588	23.025	74.8						
	2.556	4.059	1.588	22.900	76.4						
	2.554	4.056	1.588	22.915	77.7						

All values are obtained using He as pressure transmitting medium. The pressures measured with the ruby fluorescence method are all reported in GPa. The lattice parameters are reported in Å. Experimental uncertainty on lattice parameters is lower than 0.003 Å. Uncertainty on pressure measurement increases from 0.05 GPa at 1 GPa to 2 GPa at 150 GPa.

**Figure 1:**
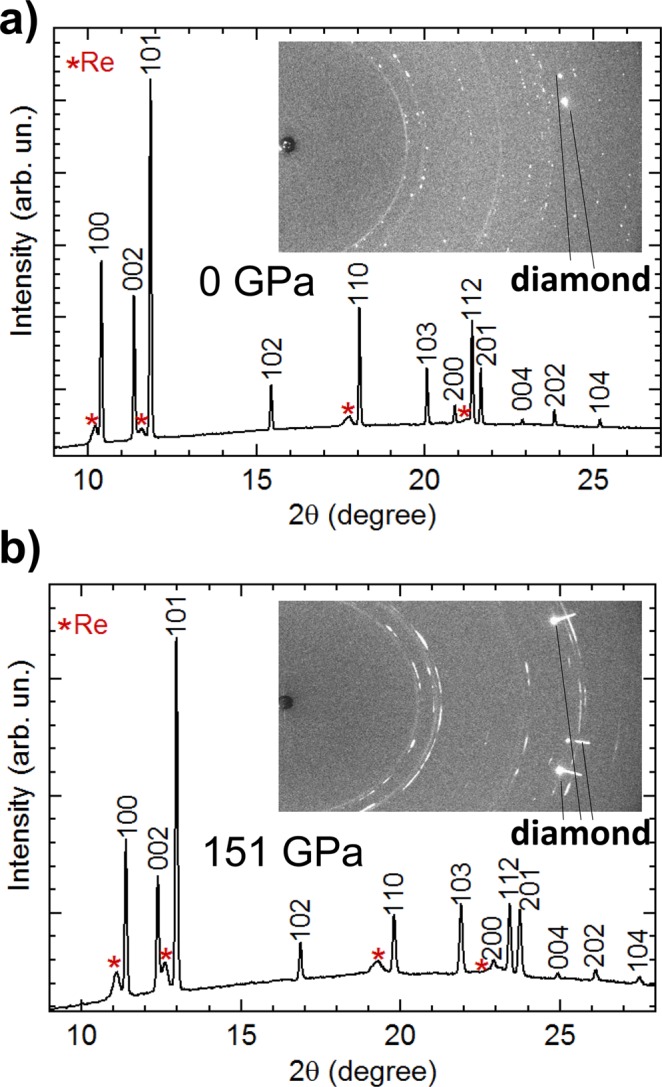
Integrated powder X-ray diffraction patterns of Re at low (**a**) and high (**b**) pressure. The insets show a part of the recorded raw diffraction images. The diamond contribution to the X-ray raw patterns are masked during the integration.

During Run2 the pressure was increased rapidly up to 92.2 GPa and a finer pressure step was then used until reaching 151 GPa, the highest pressure covered in the present experiment. Under the present experimental conditions, Ru maintains its *hcp* phase. Figure 1 shows the raw and the integrated AD-XRD patterns of Ru collected at the lowest and the highest pressure achieved at ambient temperature. In both cases, it is possible to observe the 100, 002, 101, 102, 110, 103, 112, 201, 004, 202 and 104 reflections belonging to the *hcp* structure of Ru. Few parasitic reflections coming from the Re gasket are also visible (labelled with red asterisks), this is caused by the X-ray beam tails obtained on the first table of I15. However, the quality of the Ru XRD patterns is not affected by the presence of the signal from Re.

At ambient pressure, the raw XRD image shows a signal coming from a sample composed of multiple crystals of Ru, and from a Re foil. At the highest pressure achieved (151 GPa), the signal from the Re foil is similar to the one obtained at ambient pressure, whereas, the XRD of Ru shows the formation of some texturing, with small broadening in the diffraction peaks. This behavior is typically associated with the formation of micro-stress in the sample^26^. A Williamson-Hall analysis of our data indicates that the *ξ* parameter^27^, which is a measure of peak broadening due to strain, changes from 0.5(1) × 10^−3^ at ambient pressure to 0.6(1) × 10^−3^ at 151 GPa, indicating that strains due to deviatoric stresses are small in our experiments up to the highest pressure. No evidence of structural transitions or distortions have been observed at *RT* up to 151 GPa.

A qualitative analysis of the hydrostatic conditions of the sample has been performed by comparing the measured d-spacing of Ru at the highest pressure reached (151 GPa) to the theoretical (hydrostatic) one calculated using the lattice parameter obtained by a refinement of the entire XRD pattern^26^. Table 2 shows the interplanar spacing measured at the highest pressure reached in this experiment. They deviate from the spacing calculated using the lattice parameters obtained by the refinement of the whole XRD pattern by less than 0.008%. This is within the experimental error of the present experiment. Therefore, we can conclude that the non-hydrostatic effects are below the detection limit of the present measurements. That is in agreement with the quantification of the macroscopic non-hydrostatic stress on metals embedded in helium that reaches 0.5 GPa at 150 GPa^28^.

**Table 2:** Measured reflections for Ru at 151 GPa.

hkl	d_*m*_ (Å)	d_*calc*_ (Å)	$\frac{{{\boldsymbol{d}}}_{{\boldsymbol{m}}}-{{\boldsymbol{d}}}_{{\boldsymbol{calc}}}}{{{\boldsymbol{d}}}_{{\boldsymbol{calc}}}} \%$
100	2.1384	2.1384	0.0014
002	1.9665	1.9666	0.0030
101	1.8787	1.8787	0.0027
102	1.4475	1.4475	0.0007
110	1.2346	1.2346	0.0008
103	1.1177	1.1177	0.0018
200	1.0692	1.0692	0.0019
112	1.0456	1.0456	0.0009
201	1.0317	1.0318	0.0058
004	0.9833	0.9833	0.0030
202	0.9393	0.9394	0.0075
104	0.8934	0.8934	0.0056

*hkl* are the Miller indices of the reflection. d_*m*_ is the corresponding measured inter-planar distance measured by individual peak fitting. d*calc* is the inter-planar distance calculated by a fit of the whole diffraction pattern.

**Figure 2:**
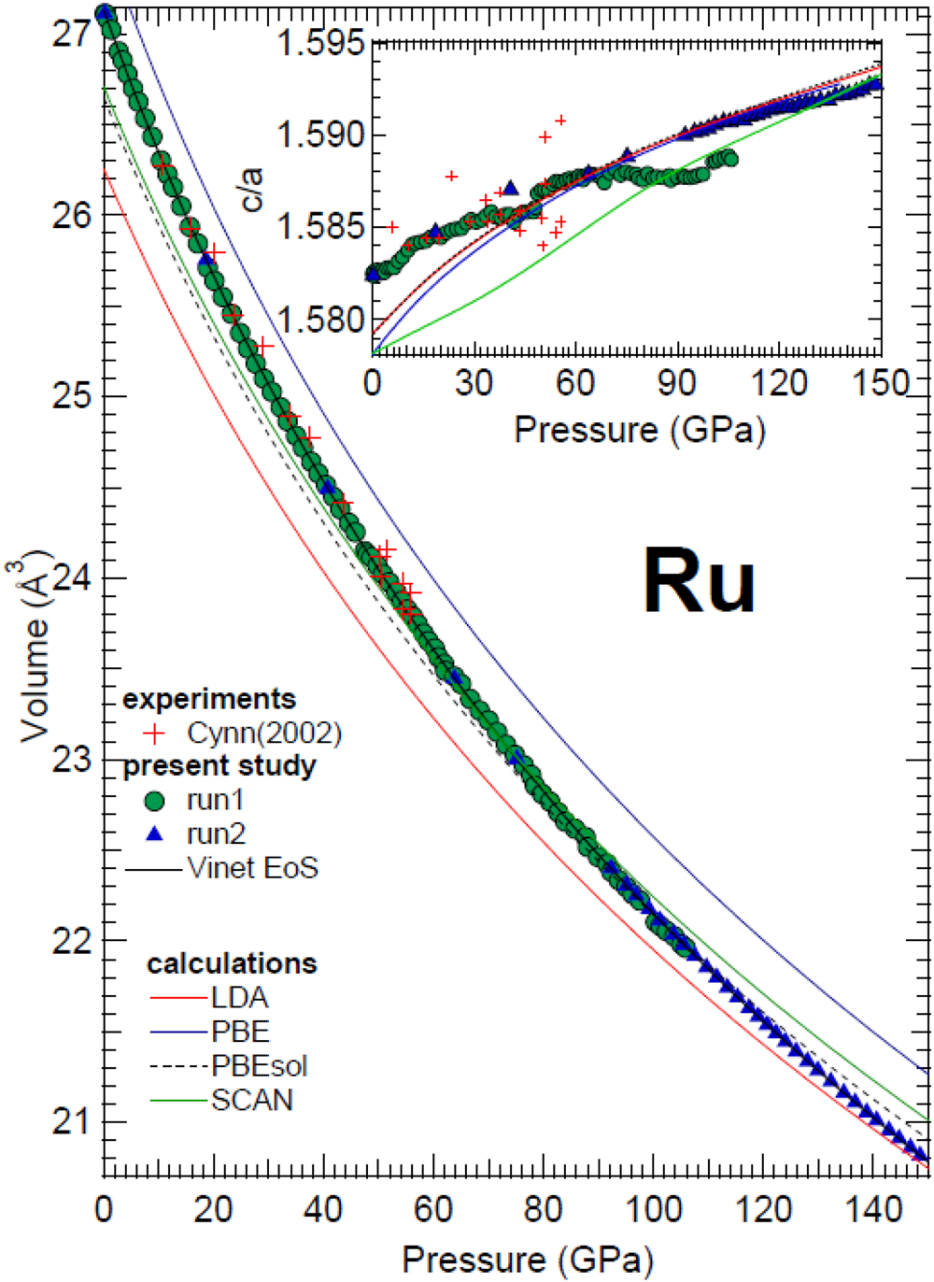
Measured and calculated volume of ruthenium as a function of pressure compared with literature data. Inset: evolution of the c/a ratio. Same symbols are used in both the main figure and the inset. In both figures the error bars are smaller than the used symbols.

In Fig. 2, the obtained compression curve is reported together with the c/a evolution as a function of pressure. The corresponding bulk modulus *K*_0_, its pressure derivative ${K}_{0}^{\text{'}}$ and the volume *V*_0_ at ambient temperature have been determined from a least-square fit of the entire set of data to a Rydberg-Vinet^29^ and a third-order Birch-Murnaghan (BM3) equation of state (EoS) using the EOSFit7c software^30^. The obtained values are reported in Table 3. The data obtained by Cynn *et al*.^22^ with an ED-XRD DAC experiment performed using Ar as pressure transmitting medium are also reported for comparison in Fig. 2. Although their data results more scattered than the present one, both compression curves show a similar trend.

**Table 3:** EOS parameters of Ru measured and calculated in different experiment.

Reference	*V*_0_ (Å^3^)	*K*_0_ (GPa), ${{\boldsymbol{K}}}_{{\bf{0}}}^{^{\prime} }$	PTM	Pressure gauge	EOS	Method
this study	27.129(4)	319.1(9), 4.40(2)	He	Ruby^51^	Vinet	AD-XRD in DAC
this study	27.122(6)	323.4(11), 4.150(19)	He	Ruby^51^	BM3	AD-XRD in DAC
^22^	27.148(6)*	348(18), 3.30(8)	Ar	Ruby^62^	BM3	ED-XRD in DAC
this study	26.25(2)	368.89(1), 4.61(1)			BM3	LDA
this study	27.53(3)	314.50(3), 4.71(2)			BM3	PBE
this study	26.65(1)	350.46(1), 4.64(1)			BM3	PBEsol
this study	26.71(1)	355.36(1), 4.67(1)			BM3	SCAN
^22^	26.276	332.8, 4.3			BM3	LDA

The volume *V*_0_, the bulk modulus *K*_0_ and its pressure derivative ${K}_{0}^{^{\prime} }$ are listed. Experimental methods and EOS formulation are specified. *Fixed parameter. PTM: Pressure transmitting medium. BM3: third order Birch-Murnaghan. ED-XRD: Energy dispersive X-ray diffraction.

In the inset of Fig. 2, it can be seen how the c/a ratio of Ru increases with pressure, showing a tendency to approach the ideal c/a value for a *hcp* structure, thus increasing the packing efficiency of the crystal structure under compression. This phenomenon is consistent with the elasticity theory^31^, according to which, for a *hcp* structure $\frac{({C}_{33}+{C}_{13})-({C}_{11}+{C}_{12})}{G}=-\,\partial \,\mathrm{ln}(c/a)/\partial \,\mathrm{ln}(V)$, where *G* is the shear modulus and *C*_*ii*_ are the elastic constants. According to the present DFT calculations, the first term of the previous equation is positive in the entire range of the investigated pressure domain. Therefore $\partial \,\mathrm{ln}(c/a)/\partial \,\mathrm{ln}(V) < 0.$ This means that the c/a ratio of Ru increases as the volume decreases (i.e. pressure increases) which is exactly what is experimentally observed here. In addition, apparent slope changes can also be observed in the experimentally determined c/a of Fig. 2. However, as the observed variations are smaller than the accuracy of the measurements, they can probably be caused by experimental artifacts. It is important to point here how the c/a variation of Ru from ambient pressure to 150 GPa is only 0.6%. This indicates that the compression of Ru occurs with little changes in its an-isotropic properties.

The c/a evolution with pressure of Ru contrasts with that of other *hcp* metals like Os^32^ and Re^17^. On one hand, Os presents a positive slope in the c/a evolution up to 150 GPa. After this critical pressure, the slope changes into a negative one. This phenomenon is due to an Electronic Topological Transition (ETT) predicted theoretically around that pressure^33^. On the other hand, Re presents a negative slope in the c/a evolution between ambient and 150 GPa^17^. This behaviour is probably caused by a recently discovered electronic transition called Core-Level Crossing (CLC)^1^. In this transition, the pressure-induced crossing of the deep 5p and 4f levels affects the valence electrons and hence the chemical bonds in the metal. This variation of chemical bonds leads to changes in the structural properties. The CLC transition cannot occurs in Ru as it does not have the 5p and 4f levels. Furthermore, the absence of anomalies in the c/a evolution in Ru rules out the presence of any other pressure-induced electronic transitions up to 150 GPa. This could be extrapolated to Technetium (Tc) as an additional *hcp* metal with a similar electronic configuration and a similar ionic radius. Regarding the bulk modulus, it is noticeable that this parameter is very similar in Re (352 GPa^17^), Ir (339 GPa^2^), and Ru (323 GPa (this work)) for experiments carried out using He as pressure medium. Being Os surrounded by these three elements in the periodic table and having a similar electronic configuration, it is then striking that it has been reported to be the material with the lowest experimentally determined compressibility (462 GPa^22^) having it a bulk modulus closer to Re, Ru, and Ir as reported from experiments performed under He (395 GPa^34^).

**Figure 3:**
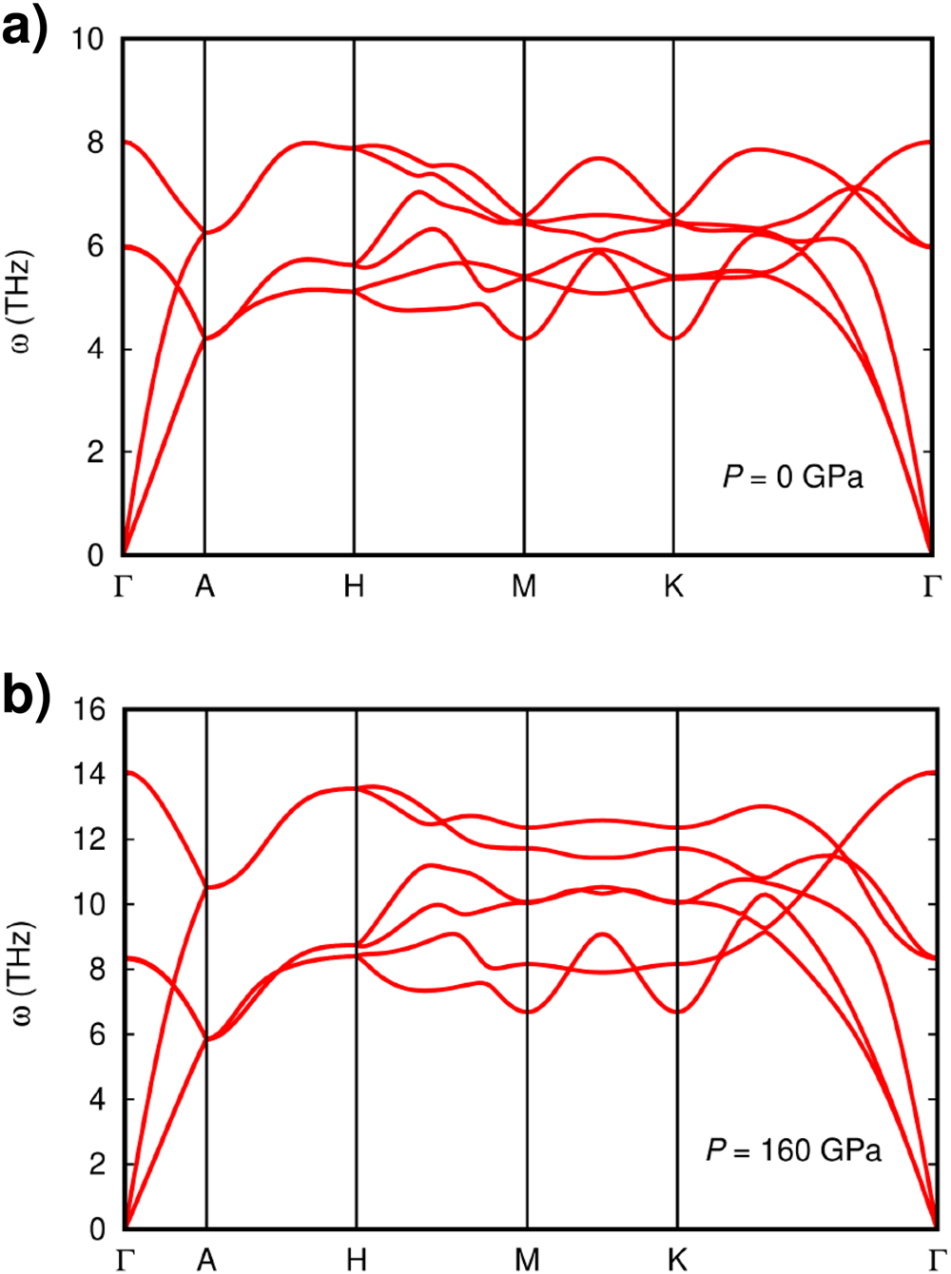
Simulated phononic dispersion curves of *hcp* Ru at ambient pressure (**a**) and at 160 GPa (**b**).

The compression curves and c/a evolution obtained in the present study by DFT using several exchange-correlation functionals, including LDA^35^, GGA-PBE^36^, GGA-PBEsol^37^, and meta-GGA SCAN^33^, are also reported in Fig. 2. In this case, the functional that displays an overall best agreement with the experiments is the GGA-PBEsol, hence most of the results that we present in this work are obtained with it. In particular, the difference between the bulk modulus experimentally measured and the one obtained via PBEsol functional is less than 10%. Furthermore, the pressure evolution of the c/a ratio is well described by the PBEsol for pressure higher than 30 GPa, confirming the anisotropic nature of the compressed *hcp* Ru. The obtained phonon dispersion at ambient and 160 GPa are shown in Fig. 3. The dispersion at ambient pressure qualitatively agrees with that reported by Heid *et al*.^38^ In particular, at ambient pressure we observe anomalies in all branches in the vicinity of the M point of the Brillouin zone, with three branches being nearly degenerate and one of them being dispersionless. Our results show that *hcp* Ru is dynamically stable up to 160 GPa. In particular, all the minima from the phonon dispersion (with the exception of the acoustic modes at Gamma) moves towards higher frequencies as pressure increase, suggesting that phonon instabilities are not expected for *hcp* Ru even beyond 160 GPa. In addition, the anomalies around M are attenuated at 160 GPa and the degeneracy between branches partially broken.

**Figure 4:**
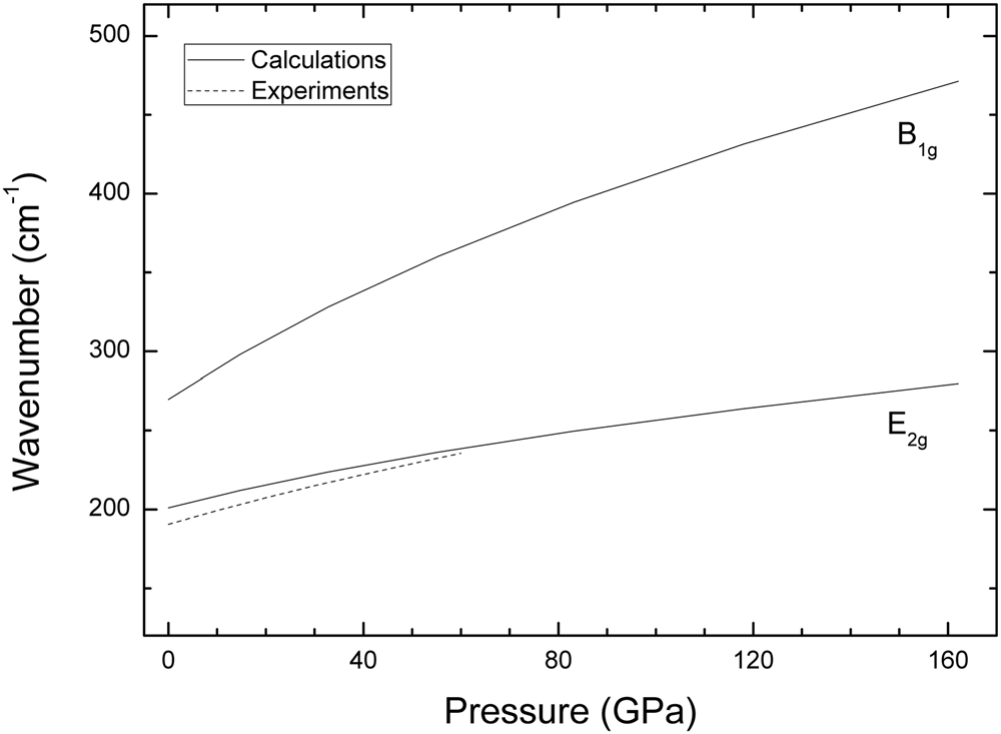
Pressure evolution of the Raman active (*E*_2*g*_) and silent (*B*_1*g*_) modes as obtained from present calculations, compared to previous work^39^.

The fact that the phonon dispersion at both pressures have all branches with positive frequencies, indicates that the *hcp* Ru is not only structurally, but also dynamically stable, *i.e*. it does not present phonon instabilities. Since the *hcp* structure has two atoms per primitive cell, it has six possible vibrations. However, at the Γ point, there are two pairs of degenerate modes (two optical and two acoustic branches). Based upon group-theory analysis, it is known that modes of the *hcp* structure at Γ are: *A*_2*u*_ + *B*_1*g*_ + *E*_2*g*_ + *E*_1*u*_. The *A*_2*u*_ mode and the degenerate *E*_1*u*_ are the acoustic modes. The degenerate *E*_2*g*_ optical mode is Raman active, and the *B*_1*g*_ optical mode is a silent mode. We have calculated the pressure dependence of the optical modes which is shown in Fig. 4. The results for the Raman-active mode agree quite well with the measurement made by Olijnyk *et al*. up to 60 GPa^39^, thus they can be considered a good prediction for the pressure dependence of the Raman mode and the silent mode up to 160 GPa. The good agreement between the quasi-harmonic calculations and the experiments is an indication that anharmonic contributions are negligible in Ru at RT. Notice that the Raman active *E*_2*g*_ mode is a shear mode in which successive hexagonal planes of the *hcp* structure beat against each other^40^. In contrast, the silent *B*_1*g*_ correspond to bending vibrations within the hexagonal plane^40^. In general, in-plane vibrational frequencies are more energetic than the out-of-plane ones. This is consistent with the results of our calculations (*B*_1*g*_ has a higher frequency than *E*_2*g*_). The fact that the *E*_2*g*_ mode is associated to an inter-planar vibration along the c-axis, makes possible to link it with the *C*_44_ elastic constant^39^; basically *C*_44_ is proportional to the square power of the frequency of the *E*_2*g*_ mode. According to this hypothesis, *C*_44_ should increase by a 60% from ambient pressure to 160 GPa, which is consistent with calculations reported by Lugovskoy *et al*.^24^ The fact that *C*_44_ is expected to have a positive pressure derivative rules out a possible phase transition from *hcp* to cubic structure.

### High pressure - high temperature evolution

**Figure 5:**
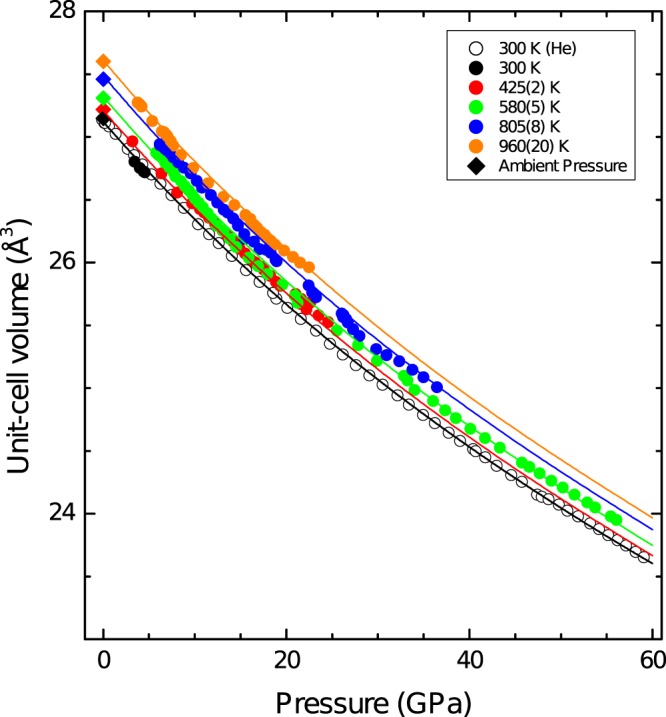
Compression curves of Ru obtained at different temperatures. The symbols represent the measured data whereas the lines represent the corresponding *P*-*V*-*T* EoS. The same color code has been used for both the experiments and the fits.

XRD experiments performed up to 56 GPa and 960 K have shown that Ru remains in the *hcp* structure with no evidence of phase transitions or structural distortions. Furthermore, the obtained data do not show any formation of Ru carbides^41^ and/or oxides^42^. The pressure and temperature dependence of the Ru unit-cell obtained from four isotherms together with the RT results are shown in Fig. 5. Also results from previous high- and room-temperature studies at ambient pressure are included^43,44^. The results from the HT experiments carried out using KCl as pressure transmitting medium are consistent with both the one obtained at room temperature using He as pressure transmitting medium and the one obtained in previous studies^43,44^. As described before, the *RT P-V* results are well represented by a third-order Birch-Murnaghan EoS^45^ with *K*_0_ = 323(1) GPa, ${K}_{0}^{^{\prime} }$ = 4.15(2) and *V*_0_ = 27.122(6) Å^3^. Regarding the HT results, the isotherms shown in Fig. 5 can be described using the Birch-Murnaghan isothermal formalism^46^. The obtained results are shown in the figure. In the fit of the P-V-T EoS the above given values for *K*_0_, ${K}_{0}^{^{\prime} }$ and *V*_0_ have been considered as fixed. The bulk modulus has been assumed to present a linear dependence from the temperature^3^:

1 $${K}_{0}(T)={K}_{0}(300)+\beta (T-300)$$

A similar functional dependence has been assumed for the thermal expansion^3^:

2 $$\alpha (T)={\alpha }_{0}(300)+{\alpha }_{1}(T-300)$$

being *α*_0_, *α*_1_ and *β* the only free parameters in the fit. As it can be seen in Fig. 5, the used approximations are sufficient for describing the pressure and temperature dependence of the volume in the *P-T* range explored in this study. The obtained values for the fitting parameters are *α*_0_ = 2.2(1) 10^−5^ K^−1^, *α*_1_ = 7.5(5) 10^−9^ K^−2^ and *β* = −1.1(2) 10^−2^ GPa/K. These values are comparable with those reported for the *hcp* phase of Ti and Zn in a similar temperature range^47,48^. The fact that a linear dependence in *T* for the thermal expansion and bulk modulus accounts well for the experimental results up to 960 K indicates that Ru behaves quasi-harmonically up to this temperature.

## Conclusion

In this work the stuctural and chemical evolution of Ru has been studied under *HP-HT* conditions combining AD-XRD and DAC techniques. The data have been collected along five different isotherms to a maximum of 151 GPa at ambient temperature and 21 GPa at 960 K, respectively. The collected data allowed a thermal EoS to be determined following a Birch-Murnaghan formalism. In the investigated *P-T* domain, only the *hcp* phase of Ru has been observed. The ambient temperature data have been obtained under quasi-hydrostatic conditions using He as pressure transmitting medium. First principle simulations based on DFT have been performed using different exchange correlation functionals to better constrain the structural and vibrational properties of Ru at ambient temperature. The PBEsol functional provided the best agreement with the experimental data when considering the ambient temperature EoS and the pressure dependence of the c/a ratio. The obtained vibrational modes confirm *hcp* Ru to be structurally and dynamically stable up to at least 160 GPa.

## Methods

### Experimental

In this work, two series of AD-XRD experiments were performed: one at the Materials Science and Powder Diffraction beamline BL04 at the ALBA synchrotron^49^, and one at the Extreme Conditions beamline I15 at the Diamond Light Source (DLS) synchrotron^50^. For the experiment performed at DLS, two membrane DACs were equipped with diamonds with culet sizes ranging from 200 to 100 *μ*m. Whereas for the experiments performed at ALBA, four membrane DACs were equipped with diamonds with culet sizes ranging from 300 to 200 *μ*m. In both cases, gaskets where prepared from pre-indented and sparkle-erosion drilled Re foils. Sample loadings were performed under an inert atmosphere to prevent sample oxidation or other possible chemical reactions. For the experiment performed at DLS, few grains of Ru (approximately 4 *μ*m each; 99.999% purity, Sigma Aldrich) were loaded at the centre of the DAC high pressure chamber. A ruby chip was placed few *μ*m away from the sample and used as pressure gauge. Finally, the He pressure medium was loaded into the cell.

For the experiment performed at ALBA, the Ru powder was compressed into pellets using two diamond anvils. The obtained pellets were cut and loaded in the DAC high pressure chambers between two FIB-cut KCl disks. The KCl disks, acting as pressure transmitting medium as well as pressure gauges, were oven dried at 200 °C for two hours before being loaded in the DAC.

Diffraction data were collected at I15 with a monochromatic X-ray beam (*λ* = 0.4246 Å) and measured using a MAR345 area detector at a distance of 300.84 mm. During the experiment, pressure inside the high pressure chamber of the DAC was determined from the ruby luminescence method using the calibration of Dorogokupets *et al*.^51^.

At ALBA, XRD patterns were collected using an SX165 Rayonix charge-coupled device camera, with a monochromatic beam (0.4246 Å) at a sample-to-detector distance of 205.84 mm. The DACs were heated using 240-V Watlow coiled-cable heaters, which were wrapped around the outside of each DAC, and which can operate continuously at a power density greater than 4.65 W/cm^2^. These heaters are capable of inducing temperatures above 900 K in samples^52^. For temperature measurements, K-type thermocouples, with an accuracy <0.4% were attached to the piston of the DAC, close to one of the diamond anvils. The DAC was housed inside a dedicated water-cooled vacuum vessel which was evacuated using a rotary-backed turbo pump. An evacuated environment prevents oxidation of the diamonds at high temperatures and removes the effects of convective heating. Typically, vacuum pressures of ~10^−4^–10^−5^ mbar were achieved within the vessel during the course of the experiments. Water cooling ensured the vessel remained cool (approximately at room temperature (RT)) relative to the heated DAC and any thermally induced movement of the sample with respect to the x-ray beam was thus minimized. This apparatus has been successfully used in previous HP-HT AD-XRD DAC experiments at different synchrotron facilities^52–54^.

The sample pressure was determined from the XRD patterns of KCl according to the thermal EOS of Dewaele *et al*.^55^.

Before each heating run, the sample was pressurized at RT to approximately 3 GPa and then heated up while collecting XRD data. Once the target temperature was reached, isothermal compressions were performed, and XRD patterns were collected every 1–2 GPa.

In both cases, the detector geometry was calibrated with a LaB_6_ standard using the powder calibration routine of the DIOPTAS software suite^56^. Masks were applied to the raw diffraction images on a per image basis before they were azimuthally integrated in DIOPTAS. The obtained diffraction data were analysed by Le Bail fitting using the routines in TOPAS software suite^57^, literature values for the lattice parameters of Ru were used as starting point for these refinements.

### Computational methods overview

First-principles calculations based on density functional theory (DFT) have been performed to analyze the equation of state and structural and vibrational properties of *hcp* Ru under pressure. The calculations were performed with the VASP code^58^ by using projector augmented-wave (PAW) method potentials^59^. Four different approaches have been tested in the simulations: Local-density approximation (LDA), generalized-gradient approximation with Perdew-Burke-Ernzerhof (GGA-PBE) and Perdew-Burke-Ernzerhof for solids (GGA-PBEsol) functionals, and meta-GGA with strongly constrained and appropriately normed semilocal functionals (SCAN). The electronic states 4*p*^6^5*s*^1^4*d*^7^ are considered as valence. Wave functions are represented in a plane-wave basis truncated at 650 eV. By using these parameters and dense **k**-point grids of 16 × 16 × 12 for integration within the first Brillouin zone (IBZ), energies are converged to within 1 meV per formula unit. In the geometry relaxations, a tolerance of 0.01 eV $\cdot$ Å^−1^ is imposed in the atomic forces. *Ab initio* phonon frequencies are calculated with the small-displacement method^25,60^ in order to assess the vibrational stability of highly compressed *hcp* Ru and estimate the *P*-dependence of the corresponding Raman mode. In the small-displacement approach, the force-constant matrix is calculated in real-space by considering the proportionality between atomic displacements and forces. The quantities with respect to which the phonon calculations are converged include the size of the supercell, size of the atomic displacements, and numerical accuracy in the sampling of the IBZ. The settings providing a quasi-harmonic free energies converging to within 5 meV per formula unit are the following: 4 × 4 × 3 supercells (where the figures indicate the number of replicas of the unit cell along the corresponding lattice vectors), atomic displacements of 0.02 Å, and **k**-point grids of 4 × 4 × 4. The value of the phonon frequencies are obtained with the PHON code developed by Alfè^61^. In using this code, the translational invariance of the system is exploited to impose the three acoustic branches to be exactly zero at the center of the Brillouin zone, and apply central differences in the atomic forces.

## Data Availability

The datasets generated during and/or analysed during the current study are available from the corresponding author on reasonable request.
