# Author Correction: Thermal equation of state of ruthenium characterized by resistively heated diamond anvil cell

**DOI:** 10.1038/s41598-020-63865-0

**Published:** 2020-04-22

**Authors:** Simone Anzellini, Daniel Errandonea, Claudio Cazorla, Simon MacLeod, Virginia Monteseguro, Silvia Boccato, Enrico Bandiello, Daniel Diaz Anichtchenko, Catalin Popescu, Christine M. Beavers

**Affiliations:** 1 Diamond Light Source Ltd., Harwell Science & Innovation Campus, Diamond House, Didcot, OX11 0DE UK; 2 Departamento de Física Aplicada - Instituto de Ciencia de Materiales, Matter at High Pressure (MALTA) Consolider Team, Universidad de Valencia, Edificio de Investigación, C/Dr. Moliner 50, Burjassot, 46100 Valencia Spain; 3 School of Materials Science and Engineering, University of New South Wales Sydney, Sydney, New South Wales 2052 Australia; 4 AWE, Aldermaston, Reading, RG7 4PR United Kingdom; 5 SUPA, School of Physics and Astronomy, and Centre for Science at Extreme Conditions, The University of Edinburgh, Edinburgh, EH9 3FD United Kingdom; 6 Institut de Minéralogie, de Physique des Matériaux, et de Cosmochimie (IMPMC), Sorbonne Université - UPMC, UMR CNRS 7590, Muséum National d’Histoire Naturelle, IRD UMR 206, F-75005 Paris, France; 7 CELLS-ALBA Synchrotron Light Facility, 08290 Cerdanyola, Barcelona Spain

Correction to: *Scientific Reports* 10.1038/s41598-019-51037-8, published online 08 October 2019

This Article contains a typographical error in the Acknowledgements section.

“S.B. has received founding from the European Research Council (ERC) under the European Union’s Horizon 2020 research and innovation program (grant agreement No 670787).”

should read:

“S.B. has received founding from the European Research Council (ERC) under the European Union’s Horizon 2020 research and innovation program (grant agreement No 724690).”

